# PTX Instructs the Development of Lung-Resident Memory T Cells in *Bordetella pertussis* Infected Mice

**DOI:** 10.3390/toxins13090632

**Published:** 2021-09-08

**Authors:** Julie Tomas, Yoon Koo, Dimitri Popoff, Vilma Arce-Gorvel, Sean Hanniffy, Jean-Pierre Gorvel, Cyrille Mionnet

**Affiliations:** 1 Centre d'Immunologie de Marseille-Luminy (CIML), Aix-Marseille University, UM2, Institut National de la Santé et de la Recherche Médicale (INSERM), U1104, Centre National de la Recherche Scientifique (CNRS), UMR7280, Parc Scientifique et Technologique de Luminy, Case 906, 13288 Marseille, France; tomas@ciml.univ-mrs.fr (J.T.); k.yoon21@gmail.com (Y.K.); popoff@ciml.univ-mrs.fr (D.P.); arce@ciml.univ-mrs.fr (V.A.-G.); hanniffy@ciml.univ-mrs.fr (S.H.); gorvel@ciml.univ-mrs.fr (J.-P.G.); 2 Laboratoire Adhesion & Inflammation, UMR INSERM 1067, UMR CNRS 7333, Aix-Marseille Université Case 937, CEDEX 09, 13288 Marseille, France

**Keywords:** *Bordetella pertussis*, pertussis toxin, resident memory T cells, lung, mouse

## Abstract

Whooping cough is a severe, highly contagious disease of the human respiratory tract, caused by *Bordetella* *pertussis*. The pathogenicity requires several virulence factors, including *pertussis* toxin (PTX), a key component of current available vaccines. Current vaccines do not induce mucosal immunity. Tissue-resident memory T cells (Trm) are among the first lines of defense against invading pathogens and are involved in long-term protection. However, the factors involved in Trm establishment remain unknown. Comparing two *B.* *pertussis* strains expressing PTX (WT) or not (ΔPTX), we show that the toxin is required to generate both lung CD4^+^ and CD8^+^ Trm. Co-administering purified PTX with ΔPTX is sufficient to generate these Trm subsets. Importantly, adoptive transfer of lung CD4^+^ or CD8^+^ Trm conferred protection against *B. pertussis* in naïve mice. Taken together, our data demonstrate for the first time a critical role for PTX in the induction of mucosal long-term protection against *B. pertussis*.

## 1. Introduction

Whooping cough (pertussis) is a vaccine-preventable infectious disease caused by the gram-negative bacterium *Bordetella pertussis* responsible of approximately 100,000 child deaths each year worldwide [1]. The pathogenicity requires several virulence factors, which act in concert to facilitate its adherence, survival and proliferation in the respiratory tract. Among them, pertussis toxin (PTX) has been shown to contribute primarily to the severity of the disease [2]. Although ptx genes are found in closely related *Bordetella* species, PTX is exclusively produced by *B. pertussis* [3]. PTX is a member of the ADP-ribosylating toxin family leading to accumulation of cAMP in target cells [4]. Depending on the target cells, these disturbances may result in a variety of biological activities, including lymphocytosis [5]. Concomitantly, PTX activates innate immune responses and modulates adaptive immune responses towards a mixed Th1/Th17 balance [6,7,8]. For all these reasons, pertussis toxin (PTX) is the major antigen for establishing an immune response against this bacterium and thus the main and common component of all current pertussis vaccines.

The objective of vaccination is to enable the individual to develop specific active protection against a microbe, using the natural resources of anti-infectious immunity to generate so-called “memory” immunity. Thus, on their first encounter with pathogens, naïve T (Tnv) cells can differentiate into several types of T cells known as “memory” cells. These memory subtypes can be phenotypically separated into central memory (Tcm), effector memory (Tem) and resident memory (Trm) T cells [9,10,11,12]. Whereas Tem and Tcm migrate almost exclusively through blood and lymphoid tissue, Trm are restricted to non-lymphoid tissues, such as the lung, brain, skin and vaginal mucosa [9,13,14]. Trm can be defined by the expression of CD69, an inhibitor of S1PR1 function, that prevents cells from exiting the tissue [15,16]. Epithelial Trm can in addition be defined by the expression of CD103, the αE subunit of the αE-β7 integrin that is required to dock these cells to E-cadherin-expressing epithelial cells [17,18,19]. However, the factors regulating the generation and maintenance of Trm, and their precise characteristics and molecular imprints within non-lymphoid tissues, are not clearly understood. If they generate good systemic immunity (Tem, Tcm), current vaccines induce little or no mucosal immunity (Trm). This may therefore explain why pertussis persists as an endemic disease even in vaccinated populations. Indeed, several studies showed that protective immunity against *B. pertussis* wanes rapidly after immunization of children with current acellular vaccines (aP) [20,21]. Moreover, these vaccines do not prevent nasal colonization or transmission of the bacteria [22,23]. Recently, the work of KHG Mills and colleagues demonstrated that in contrast to what observed in mice immunized with whole-cell *pertussis* (wP) vaccines, immunization of mice with an aP failed to generate CD4^+^ TRM cells or to protect against nasal colonization as well as lung infection with *B. pertussis* [24]. In addition, numerous animal infection models have demonstrated the control of secondary infection by Trm [13,17]. This strategic tissue positioning gives Trm an enhanced capacity to fight reinfection over circulating memory T cells [25]. Thus, due to their strategic positioning there is much interest in how to optimize the local differentiation and effector function of Trm cells in order to protect peripheral barrier sites from recurrent infection.

By comparing two *B. pertussis* strains producing PTX (WT) or not (ΔPTX), we demonstrate, for the first time, a pivotal role for a toxin in generating lung-resident memory T cell populations (CD4^+^, CD8^+^ and γδ), which expand upon recall. The most frequently generated Trm are IL-17-producing cells, which favor bacterial clearance and are localized in the lung epithelial layer, a privileged colonizing site for the bacteria. Significantly, co-administration of purified PTX with ΔPTX restored the ability to generate these Trm populations during infection.

## 2. Results

In this study, we aimed at deciphering the impact of pertussis toxin (PTX) on the immune response during *B. pertussis* infection by comparing the T cell-mediated immunity triggered by a PTX producing strain (WT) to that of a PTX-deficient, isogenic strain (ΔPTX) [26].

### 2.1. PTX Is Required to Generate CD69^+^CD103^+^ T Cell Populations

**Figure 1:**
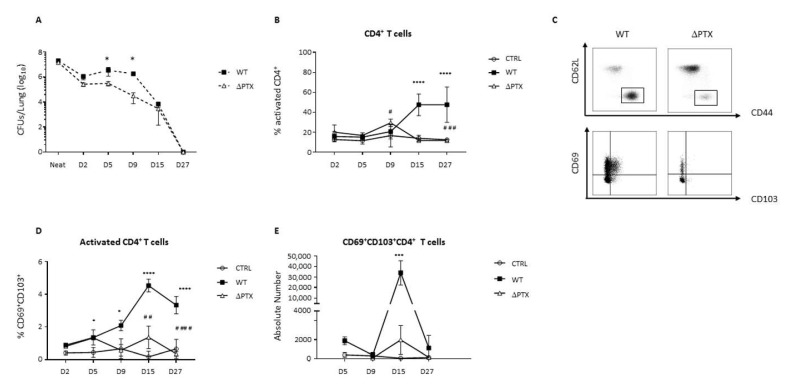
Pertussis Toxin (PTX) is required to generate CD69^+^CD103^+^ T cell subsets in the lung of primary infected mice. (**A**) Numbers of colony forming units (CFU) of *B. pertussis* strain producing PTX (WT) or not (ΔPTX) in primary infected mice enumerated at various times post infection (p.i). Mice were intra-nasally inoculated with 6 × 10^6^ CFU/20 µL of WT or ΔPTX or with 20 µL of PBS (CTRL group). (**B**) Frequency of activated CD4^+^ T cells. (**C**) Gating strategy, frequency (**D**) and absolute numbers (**E**) of CD4^+^ CD69^+^CD103^+^ recorded in the lung from D2 to D27 p.i. in primary infected mice. Results are mean ± SD for at least 4 to 5 mice/group and are representative of three independent experiments. Statistical significance of differences are shown. * *p*-value < 0.05 WT vs. CTRL; *** *p*-value < 0.001 WT vs. CTRL, **** *p*-value < 0.0001 WT vs. CTRL; ## *p*-value < 0.01 WT vs. ΔPTX, ### *p*-value < 0.01 WT vs. ΔPTX, and #### *p*-value < 0.0001 WT vs. ΔPTX.

Both strains colonized the lung of intra-nasally infected mice [26,27]. However, at days 5 and 9 post-infection (p.i.), WT-infected mice contained significantly more colony-forming units (CFU) in their lungs than ΔPTX-infected mice (Figure 1A), confirming that PTX improves lung colonization by *B. pertussis* during the early time points p.i [2,26,28]. Thereafter, there was a progressive clearance of both strains leading to their complete elimination at day 27 p.i. (Figure 1A). T cell responses are critical for protection against *B. pertussis* [8,29,30]. As shown in Figure 1B, the frequency of CD62L^−^CD44^+^CD4^+^ T cells (activated CD4^+^) was higher at day 9 p.i for the ΔPTX group than the WT group, confirming the role of PTX in delaying the adaptive immune response. However, from day 9 onwards the WT group exhibited a significant increase in the frequency of activated T cells, in contrast to the ΔPTX group (Figure 1B). In order to decipher the different subsets of T cells involved in immune response against *B. pertussis*, we phenotypically characterized the T cells by flow cytometry. As shown in Figure 1C–E, we observed a strong expansion of specific subsets expressing CD69 and/or CD103 specifically in the lung of the WT group (Figure S1). This was first evident at day 9 p.i., reaching more than 4% of active CD4^+^ T cells at day 15 p.i. and maintained thereafter up to day 27 p.i. (Figure 1D,E) when all bacteria were cleared (Figure 1A). These CD69^+^CD103^+^ subsets were much less induced in the ΔPTX group and not maintained (Figure 1D,E).

To date, most studies concerning tissue resident lymphocytes responding to respiratory infection have focused on CD8^+^ T cells subpopulation [15,31,32,33]. For this reason and despite indications of an induction of CD69^+^CD103^+^ CD4^+^ T cells during wild-type *B. pertussis* infection [17], we also investigated the presence of their CD8^+^ T cells counterpart in our experimental groups. Concomitantly to CD69^+^CD103^+^ CD4^+^ T cells, we identified, to a lesser extent (7 times less compared to CD4^+^Trm), CD69^+^CD103^+^ CD8^+^ T cells in the groups firstly exposed to WT (Figure S2A). During primary infection with WT, the number of CD69^+^CD103^+^ CD8^+^ T cells reached the highest value at day 15 p.i. and a pool of cells was maintained at day 27 p.i. In contrast, this subset was not detected in the ΔPTX groups (˂100 cells) (Figure S2A).

These CD69^+^CD103^+^ subsets (CD4^+^ and CD8^+^) have all the hallmarks of tissue-resident memory cells (Trm). Of note, γδ and CD4^+^ Trm cells were recently described to express CD69 and CD103 in mice during *B. pertussis* infection [17,34]. Moreover, these cells were described to reside at mucosal surfaces such as the lung and were able to respond rapidly to infection in the absence of T cell recruitment from the circulation.

### 2.2. CD69^+^CD103^+^ T Cell Populations Expand upon New Bacteria Challenge

**Figure 2:**
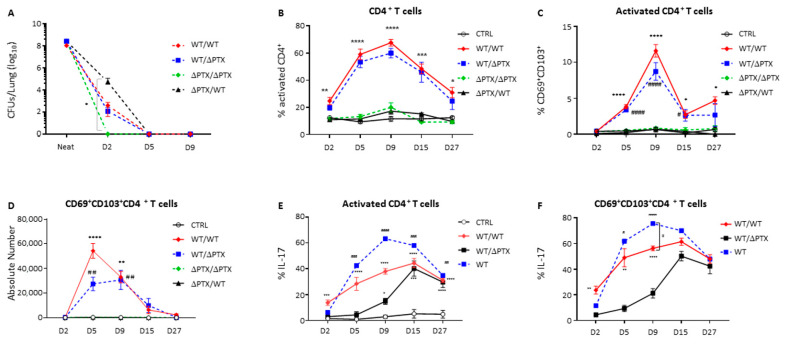
Recall infections lead to expansion of CD69^+^CD103^+^ T cell subsets in the lung of first PTX-exposed infected mice. Mice infected twice were intra-nasally inoculated with 6 × 10^6^ CFU/20 µL of WT or ΔPTX strains 6 weeks after the primary infection. (**A**) Numbers of CFU following a second infection from D2 to D9 p.i in WT/WT, WT/ΔPTX, ΔPTX/ΔPTX and ΔPTX/WT groups. Frequency of (**B**) activated CD4^+^ T cells and (**C**) CD69^+^CD103^+^ T cells, recorded from D2 to D27 p.i. in secondary infected mice. (**D**) Absolute numbers of CD69^+^CD103^+^ CD4^+^ T cells. (**E**) Frequency of lung IL-17A-producing CD4^+^activated T cells, and (**F**) CD69^+^CD103^+^ CD4^+^T cells harvested from D2 to D27 p.i. following WT, WT/WT, WT/ΔPTX or PBS (CTRL) inoculation. Results are mean ± SD for at least 4 to 5 mice/group and are representative of three independent experiments. Statistical significance of differences are shown. For Figure 2B–E * *p*-value < 0.05 WT/WT vs. CTRL, ΔPTX/ΔPTX and ΔPTX/WT; ** *p*-value < 0.01 WT/WT vs. CTRL, ΔPTX/ΔPTX and ΔPTX/WT, *** *p*-value < 0.001 WT/WT vs. CTRL, ΔPTX/ΔPTX and ΔPTX/WT, **** *p*-value < 0.0001 WT/WT vs. CTRL, ΔPTX/ΔPTX and ΔPTX/WT; ## *p*-value < 0.01 WT/ΔPTX vs. CTRL, ΔPTX/ΔPTX and ΔPTX/WT, ### *p*-value < 0.01 vs. CTRL, ΔPTX/ΔPTX and ΔPTX/WT, #### *p*-value < 0.0001 vs. CTRL, ΔPTX/ΔPTX and ΔPTX/WT. For Figure F: * *p*-value < 0.05 WT/WT vs. CTRL; ** *p*-value < 0.01 WT/WT vs. CTRL, *** *p*-value < 0.001 WT/WT vs. CTRL, **** *p*-value < 0.0001 WT/WT vs. CTRL; ## *p*-value < 0.01 WT/ΔPTX vs. CTRL, ### *p*-value < 0.01 vs. CTRL, amd #### *p*-value < 0.0001 vs. CTRL.

To confirm the memory phenotype of our PTX-induced CD69^+^CD103^+^ T cells, we tested whether an enhanced protection was observed subsequently to a re-infection. Groups of mice previously inoculated with WT or ΔPTX were re-challenged by homologous or heterologous inoculation, defining 4 different groups: WT/WT, WT/ΔPTX, ΔPTX/ΔPTX and ΔPTX/WT. In all re-challenged groups, bacteria were cleared within 5 days (Figure 2A). A time-course analysis of ongoing immunity showed an immediate T cell response upon infection, associated with an acute increase of CD4^+^activated T cells in groups first infected with WT (WT/WT and WT/ΔPTX) (Figure 2B). Most importantly, we found that among activated CD4^+^ T cells, there was a rapid increase in the CD69^+^CD103^+^ subset from day 5 until day 9 p.i only in groups having first received WT. In this subset, CD4^+^ activated cells were maintained at around 5% up to day 27 p.i (Figure 2C). These data confirm that the induction of the CD69^+^CD103^+^ subset depends on a first exposure to PTX. However, this subset, which bears the hallmark of a tissue resident memory population, was not only PTX-specific due to its accelerated expansion upon reinfection with ΔPTX (Figure 2C,D).

More, during the recall experiments, only the WT/WT group exhibited an expansion of the CD69^+^CD103^+^ CD8^+^ T cells from day 2 p.i. to reach the highest value at day 15 p.i. (Figure S2B). Taken together, these data underline the requirement of PTX to generate lung CD69^+^CD103^+^ memory (CD4^+^, CD8^+^) T cell populations, with the hallmarks of a resident phenotype that expands upon recall (Figure 1 and Figure 2).

### 2.3. Trm Are Localized at the Front Line of Defense

**Figure 3:**
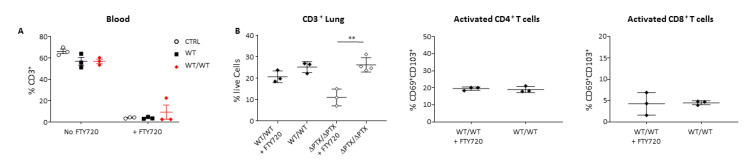

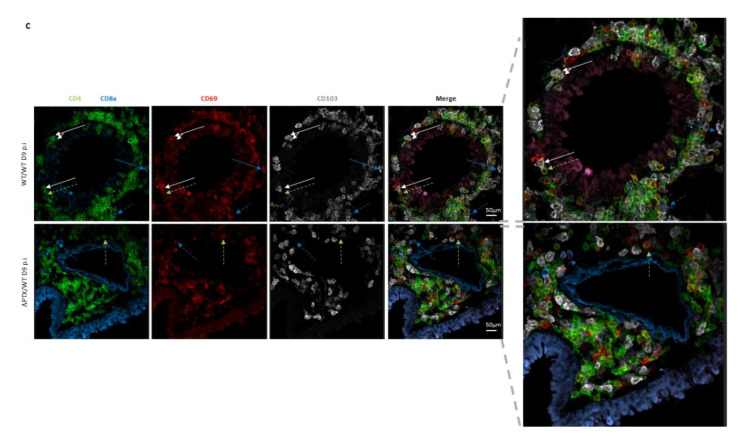
Residency characterization of the CD4^+^/CD8^+^ Trm cell subsets in the lung of infected mice. (**A**) Frequency of CD3^+^ T cells harvested from the blood after or not treatment with FTY720 in WT, WT/WT and CRTL groups at day 5 p.i. (**B**) Frequency of CD3^+^ T cells and CD4^+^/CD8^+^ Trm cells harvested from the lung after or not treatment with FTY720 in WT/WT group at day 5 p.i. (**C**) Representative micrographs of CD4^+^/CD8^+^ Trm cells taken by confocal microscopy, at day 9 pi. in WT/WT and ΔPTX/WT infected mice. Section stained for CD4 (green), CD8 (cyan), CD69 (red) and CD103 (grey). Arrows indicate CD4^+^ (green) and CD8^+^ (blue) T cells. White arrows indicate CD4^+^ or CD8^+^ Trm. Results are mean ± SD for at least 3–4 mice/group and are representative of three independent experiments. Statistical significance of differences are shown. ** *p*-value < 0.01.

The tissue residency characteristics of these subsets were more closely examined in the WT/WT-group. We treated infected mice with the fingolimod FTY720 and lymphocyte egress was examined (Figure 3A). As expected, due to the high frequency of CD69^+^CD3^+^ T cells in the WT/WT group, more CD3^+^ T cells were sequestered in the lung as compared to ΔPTX/ΔPTX group upon FTY720 treatment (Figure 3B). The CD69^+^CD103^+^ CD4^+^ and CD8^+^ T cells were maintained in the tissue during FTY720 treatment (Figure 3B) and proliferated during the course of infection (Figure S3A). The migratory properties of Trm allowed them to localize and expand at the front line of defense upon re-infection. While micrographs have previously shown CD8^+^ T cells to be associated with the parenchyma of tissues close to the airways and large blood vessels during *B. pertussis* infection [31], the in-situ localization of Trm (CD4^+^ and CD8^+^) in the lung has yet to be demonstrated. Using confocal microscopy, we found that CD69^+^CD103^+^CD4^+^ and CD69^+^CD103^+^CD8^+^ T cells localized to the epithelial layer surrounding large airways during infection (Figure 3C). Moreover, and in accordance with their kinetics of induction, there was a higher proportion of these cells present in the WT and WT/WT groups (at day 15 (data not shown) and at day 9 p.i., respectively). Despite the fact that we found very few CD8^+^ T cells within the lung of WT or ΔPTX infected mice, CD69^+^CD103^+^CD8^+^ T cells were mostly localized within the lung parenchyma close to airways (Figure 3C). Using FISH technology, we also observed high frequencies of WT bacteria localized within the bronchial lumen and attached to the ciliated epithelium in the proximity of the CD69^+^CD103^+^CD4^+^ T cells (Figure S3B). Taken together, these data confirm the tissue resident memory phenotype of our CD69^+^CD103^+^ T cell subsets (CD4^+^ and CD8^+^) (Figure 3) at the front line of defense against *B. pertussis*.

### 2.4. Trm Favor a Th17 Environment in the Lung of Infected Mice

While IL-4^−/−^ mice are able to clear bacteria as fast as the wild-type mice, suggesting that Th2 is dispensable, IL-17^−/−^or IFN-γ^−/−^ mice are unable to do so, indicating the critical role of the Th1/Th17 immune responses in protection against *B. pertussis* [7]. CD4^+^ T cells expanding locally during *B. pertussis* infection have been shown to generate a predominant Th17 microenvironment [6,7,8]. As shown in Figure 2E,F, IL-17 production by activated CD4^+^ T cells and CD4^+^ Trm significantly increased during infection with WT. The frequency of IL-17^+^ CD4^+^ cells reached maximum levels at day 15 p.i. for the WT and WT/WT groups, and at day 9 p.i. for the WT/ΔPTX group, these time points corresponding to when the highest numbers of CD4+ Trm were observed during primary or secondary infection (Figure 1D and Figure 2C,D). Interestingly, more than 70% and 50% of CD4^+^ Trm produced IL-17 in the WT/ΔPTX and WT/WT groups, respectively (Figure 2F), highlighting the key contribution of this Trm population to producing a Th17 environment during infection. As expected, the kinetics of induction of IL-17 production and expansion of Trm directly coincided with the clearance of bacteria in each of the relevant groups (Figure 1A and Figure 2A). There was also an increase in IL-17-producing CD8^+^ Trm but again to a lesser extent than the CD4^+^ Trm (no more than 20% of CD8^+^ Trm were IL-17-producing) (Figure S2B). Interestingly, the magnitude of the CD8^+^ Trm response was identical during the primary or the secondary infection (Figure S2A,B), as opposed to the significant 2-fold increase response of CD4^+^ Trm at recall (Figure 2C). In contrast to IL-17, the IFN-γ profile of the CD4^+^ T cells showed no significant differences between the groups (Figure S4A). However, shortly after the infection (day 2 p.i.), 40% of CD4^+^ Trm produced IFN-γ in the WT and WT/WT groups and then halved and maintained around 20% from day 5 p.i. onwards (Figure S4B). The same trend was observed for IL-4 and IL-2 with the difference that on day 2 p.i. more than 90% of CD4^+^ Trm produced IL-4 (Figure S4B). Almost 100% of the CD4^+^ Trm produced TNF-α during the entire course of the experiments (Figure S4B). This correlates with the induction of IL-17 and the inflammatory Th17 environment linked to exposure to PTX. In contrast, enhanced induction of IL-2 and IL-4 in the ΔPTX/ΔPTX and WT/ΔPTX groups, at day 9 p.i, suggests an early effector response that is predominantly Th1/Th2 rather than Th17 in the absence of PTX exposure. The profile of the WT/ΔPTX group seems to be a combination of the three responses Th1/Th2/Th17 in the absence of PTX during the recall phase (Figure S4A). Together, these results show that wild-type *B. pertussis* infection does not further favor Th1 to Th2 responses but rather reinforce the notion that protection is based on a predominant Th17 environment, in which CD4^+^ and CD8^+^ Trm are major contributors by producing IL-17 upon PTX exposure.

### 2.5. PTx Is Critical to Generate Trm

**Figure 4:**
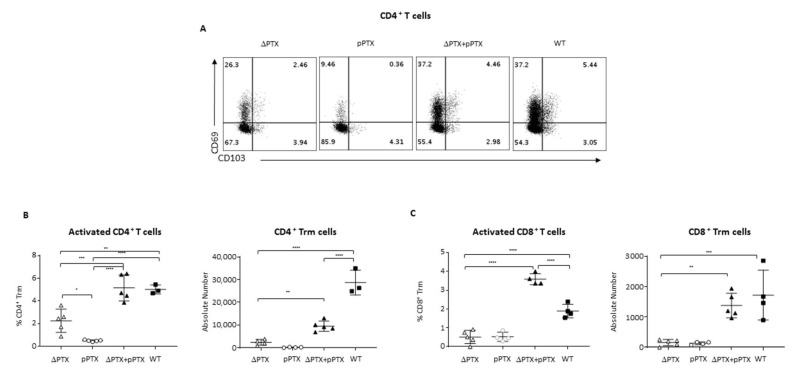
The generation of Trm cells is dependent on PTX exposure during *B. pertussis* infection. (**A**) Representative flow cytometry of CD69 and CD103 expression of CD62L^−^CD44^+^CD4^+^ T cells harvested from lung in ΔPTX, pPTX, ΔPTX + pPTX and WT groups at day 15 p.i. Frequency and absolute numbers of (**B**) CD4^+^ Trm and (**C**) CD8^+^ Trm harvested in the lung of ΔPTX, pPTX, ΔPTX+pPTX and WT groups at day 15 p.i. Results are mean ± SD for at least 3 to 5 mice/group and are representative of two independent experiments. Statistical significance of differences are shown. * *p*-value < 0.05; ** *p*-value < 0.01, *** *p*-value < 0.001 and **** *p*-value < 0.0001.

To confirm that PTX is essential for generating Trm (CD4^+^, CD8^+^) in the lung, mice were inoculated with ΔPTX co-administered with purified PTX (ΔPTX + pPTx). Surprisingly, a single dose of pPTx was sufficient to generate CD4^+^ Trm in the ΔPTX + pPTX group at day 15 p.i. (Figure 4A,B). While there was notable difference in absolute numbers of CD4^+^ Trm in the ΔPTX + pPTX group compared to the WT group, CD4^+^ Trm numbers significantly increased compared to groups administered ΔPTX or pPTX alone (Figure 4B). In contrast, the number of CD8^+^ Trm generated in the ΔPTX + pPTX group was similar to that seen in the WT group (Figure 4C). As previously shown by Misiak and colleagues, γδ T cells expand during WT infection (Figure S5A–C). Surprisingly, adding pPTX to ΔPTX strain was not only sufficient to induce γδ T but also resulted in γδ Trm expansion (Figure S5C). Altogether, these data confirm that PTX is absolutely critical to generate Trm in the lung of *B. pertussis* infected mice.

The significant increase in lung colonization by ΔPTX when co-administered with pPTX (Figure S5D) would support previous studies demonstrating a role for the toxin in delaying the immune response that facilitates colonization especially during the early stages of infection [10,11]. Paradoxically, PTX-mediated inhibition of chemotaxis [35] may also enable lung retention of newly activated T cell leading to their compartmentalization and development as Trm. De novo production and secretion of PTX during the course of infection can explain the increased numbers of Trm (in terms of absolute numbers) in the WT group compared to the ΔPTX + pPTX group.

### 2.6. Trm Are Sufficient to Protect against a New Infection

**Figure 5:**
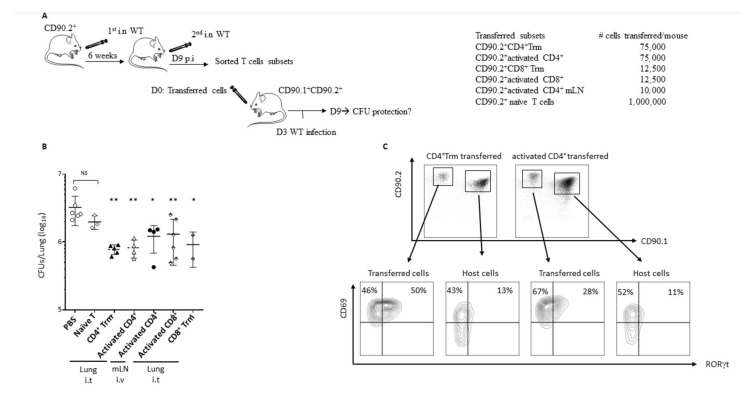
Transfer of Trm confers protection against *B. pertussis*. (**A**) Schematic representation of the adoptive transfer protocol and number of cells transferred in naïve CD90.1^+^CD90.2^+-^recipient mice. (**B**) Recipient mice were killed at day 6 p.i for the CFU protection analyses. Results are mean ± SD for at least 2 to 6 mice/group and are representative of two independent experiments. All the groups were compared to the PBS control group. Statistical significance of differences are shown. * *p*-value < 0.05; ** *p*-value < 0.01. (**C**) Representative flow cytometry panels to follow transferred and host cells. CD69 and RORγt expression of transferred and host cells harvested from lung of recipient mice at day 6 p.i. The upper right quadrant indicates the frequency of RORγt positive cells.

Adoptive transfer of CD4^+^ T cells into naïve mice has already demonstrated the importance of T-cell immunity in clearing *B. pertussis* in mice (Mills et al., 1993). To assess their role in mediating protection against PTX-producing *B. pertussis*, we adoptively transferred CD90.2^+^ Trm (CD4^+^ or CD8^+^). We used as positive controls CD90.2^+^CD62L^−^CD44^+^CD69^+^CD103^−^ CD4^+^ or CD8^+^ T cells. Each of these subsets were sorted from the WT/WT mice at day 9 p.i (Figure 5A). The CD90.2^+^CD3^+^CD62L^+^ (naïve T) subset coming from naïve mice was also investigated as a negative control. The recipient CD90.1^+^/CD90.2^+^ naïve mice once transferred were then infected with WT and protection was assessed (Figure 5A). As expected, transfer of naïve T cells did not significantly decrease the bacterial load at day 6 p.i. (Figure 5B). However, all the other transferred populations significantly reduced the bacterial burden in the lung of the mice at day 6 p.i. (Figure 5B). Transferred CD4^+^ or CD8^+^ Trm are sufficient to provide significant protection against PTX-producing WT to a similar extent to the protection conferred by activated T cells, which includes all effector T cells (Figure 5B). In our case, protection conferred by the transferred CD4^+^ and CD8^+^ Trm was probably mediated through the Th17 environment, as suggested by the ROR-γt plots (Figure 5C).

## 3. Discussion

To summarize, whooping cough is an important cause of morbidity and mortality in infants, young adults and seniors worldwide and continues to be a public health concern despite high vaccination coverage [36,37,38]. Following an increase in resurgence of the infection in developed countries using acellular vaccines, interest is now focused on mucosal memory responses generated by *B. pertussis*. In two previous studies, Mills KH et al. observed for the first-time resident memory CD4^+^ and γδ T cells in the lung of *B. pertussis* infected mice [17,34]. These cells play an important role in the protection against *B. pertussis* as they are supposed to reside physically at the first line of defense against bacteria and favor a predominant Th17 environment in the lung of infected mice. For the first time, we visualized lung CD4^+^ and CD8^+^ Trm by confocal microscopy, confirming that they can directly create a specific environment to improve immune response against a new *B. pertussis* infection (Figure 2E,F, Figure 3C, Figure 5C and Figure S5E,F). However, what triggers these populations is not known. It is well established that CD69 antagonizes S1PR1 [16], which is required for migration of T cells out of tissues; hence, S1PR1 inhibition would facilitate tissue residency [39]. Indeed, we observed a lesser frequency of activated (CD62L^−^CD44^+^) and so of CD69 expressing T cells in ΔPTX infected mice. Does the strength of inflammation increase the number of activated T cells expressing CD69 and so conversion for a part of them in Trm. To test that, we infected mice with a higher dose of *B. pertussis* ΔPTX. As shown in Figure S6, we were able to restore similar frequencies of CD44^+^ and CD69^+^ CD4^+^ T cells. However, this did not lead to the formation of CD4^+^ Trm. In the opposite, administration of a single dose of purified PTX with *B. pertussis* ΔPTX inoculum (Figure 4) can successfully (i) increase the frequency of activated CD4^+^ T cells (Figure S5C) and (ii) restore the level of generated Trm (CD4^+^, CD8^+^ and γδ). This confirms that PTX directly influences the generation of Trm either via its inhibition of chemokine receptor signaling or via a synergy with other *B. pertussis* toxins or both. Previous reports have shown that upregulation of CD103 always occurs late during the infection process in order to affect the persistence of Trm long after the cells had reached the site of infection [40]. Indeed, this is confirmed by our experiment where mice were firstly exposed to ΔPTX strain and re-challenged with the WT strain. Even in this case, we did not observe a de novo generation of Trm. This could be explained by the quick elimination of the inoculum due to other memory cells (Tem, Tcm and memory B cells). Our study leads us hypothesize that raising from the CD62L^−^CD44^+^CD69^+^CD103^−^ T cell subset, CD4^+^ and CD8^+^ differentiated into Trm (CD62L^−^CD44^+^CD69^+^CD103^+^) only if they were first exposed to PTX due to a longer retention in infected lungs. However, it can be argued that current vaccines contain PTX, but they do not induce formation of Trm [24]. This could be due to the fact that either vaccines do not contain all the *B. pertussis* antigens or because they contain a modified PTX (WHO, 2016). Considering PTX as an adjuvant, its interaction with dendritic cells has also been shown to increase the adaptive response [41] and to enhance diverse lymphocyte expansion, including Th1, Th2 and Th17 responses, while reducing the number and function of regulatory T cells [42]. In addition, the eventual absence of PTX (and its adjuvant properties) following an initial administration would minimize the impact of any response that specifically targets the toxin and reduce the magnitude of the adaptive T cell response (including Trm) that is dependent on but not necessarily specific to PTX. Moreover, Mills KHG et al. showed in vitro that lung-isolated Trm can be re-stimulated by different antigens, but they did not demonstrate any specificity or identify a particular role for any given antigen in generating resident populations. We observed the same thing in vivo as our CD4^+^Trm were able to expand after a second infection with ΔPTX strain (Figure 2B). On the other hand, in our study we observed that the generated CD8^+^ Trm seem to be PTX specific. Indeed, CD8^+^ Trm did not expand if the first WT-infected mice were re-challenged with the non-expressing PTX strain (ΔPTX) (Figure 2B). In accordance with this latter, we also observed that these CD8^+^Trm are hyper-efficient against a new infection with WT *B. pertussis* strain. Indeed, only 12,500 transferred intra-tracheally in naïve mice are sufficient to favor elimination of WT strain (Figure 5). It would be interesting to test their efficiency against a ΔPTX strain infection in naïve mouse.

In conclusion, we demonstrate in vivo that PTX is strictly required to generate lung Trms (CD4^+^, CD8^+^ and γδT cells) against *B. pertussis*. Furthermore, our hypothesis was corroborated by increased T cell responses that were observed in mice co-administered ΔPTX with purified PTX. Further studies are needed to (i) define the exact role of Trm in long-term protection and to (ii) define their ontogeny.

Finally, deciphering the pivotal role of PTX (or PTX-producing *B. pertussis*) on the Trm generation can provide a new basis for PTX-mediated immunity with implications in vaccine strategies to improve long-term protection against *B. pertussis*.

## 4. Materials and Methods

### 4.1. Bordetella Pertussis Strains and Growth Conditions

To investigate impact of pertussis toxin (PTX), we used the wild *B. pertussis* Tohama I (WT) and BpΔPTX (ΔPTX) strains. The ΔPTX strain has a deletion of the pertussis toxin operon (Alonso et al., 2001). Bacteria were grown on Bordet-Gengou blood agar plates for 4 days at 37 °C. Subsequently, multiple colonies were transferred onto new plates and grown for 2 days at 37 °C prior to experimental infection.

### 4.2. Mice

This study was carried out in strict accordance with the guide lines of the Council of the European Union (Directive 86/609/EEC) regarding the protection of animals used for experimental and other scientific purposes. The experimental protocol was approved by the institutional animal care and use committee of the Aix-Marseille University (n° 04287.01, acceptance 28 March 2017). All experiments were done in accordance with French and European guidelines for animal care. Seven-week-old females BALB/c (Janvier Laboratories; Le Genest St Isle France) were maintained under Animal Biosafety Level 2 facility and fed food and water ad libitum.

### 4.3. Infection Model

*B. pertussis* grown on Bordet-Gengou plates was suspended in ice-cold sterile PBS and adjusted to a concentration of 3 × 10^8^ CFU/mL. Primary infections were performed by the intra-nasal route with 20 μL of the bacterial suspension (6 × 10^6^ CFU) deposited in the nostrils of 7 week-old female Balb/c mice, anesthetized with 200 μL of a Rompun-Ketamin mix by peritoneal injection. Six weeks later, secondary infections were performed by the same method than previously described.

### 4.4. Fingolimod Treatment

For FTY720 experiment (item n°10006292, Cayman Chemical Company, Ann Arbor, MI, USA), mice were treated with 8 mg/mL of FTY720 in drinking water 24H before intranasal infection with *B. pertussis*. After infection, FTY720 was daily added in drinking water during the 5 days of the experiment.

### 4.5. BrdU Pulse Chase Experiment

For 5-Bromo-2′-deoxyuridine (BrdU) experiment (B500-2, Sigma-Aldrich, Saint Quentin Fallavier, France), mice were injected with 100 µL of BrdU (10 mg/mL) by peritoneal injection during infection. After infection, 667 µg/mL of BrdU were daily added in drinking water during the 5 days of the experiment.

### 4.6. Pertussis Toxin Complementation

For PTX complementary experiment, 100 ng of purified PTX (70323-44-3, TOCRIS, Noyal Châtillon sur Seiche, France) was added in ΔPTX strain culture or in PBS solution during infection.

### 4.7. Isolation of Lymphocytes

#### 4.7.1. Lungs

Lungs were removed from the mouse after perfusion of the heart with PBS. Lungs were cut into very small pieces (approximately 1–2 mm^3^) in gentleMACS C Tubes (MiltenyiBiotech, Paris, France) containing 3 mL of digestion solution (DNASE I 10 mg/mL (Sigma-aldrich, Saint Quentin Fallavier, France) + Collagenase II 70 mg/mL (Worthington, LS 004176, Serlabo technologies, Entraigues sur la Sorgue)) in 5% RPMI medium. Lungs were cut into smaller pieces with the GentleMACS dissociator machine (10 s, MiltenyiBiotech). The lungs were digested at 37 °C for 30 min under constant horizontal shaking (200 rpm) and then again grinded in the dissociator machine (45 s, MiltenyiBiotech, Paris, France). Stop buffer (1X PBS, 0.1 M EDTA) was added before filtering the lung digestion on 100 µm strainer. With the help of a sterile syringe, the remaining lung pieces was push through the sieve. The sieve was washed with 5 mL RPMI/5% FCS, and the lung digestion was centrifuged at 400× *g* for 10 min. The pellet were resuspended in 1 mL of red blood cell (RBC) lysis buffer (Sigma-Aldrich, Saint Quentin Fallavier, France) and incubated for 2 min at room temperature (RT). Nine mL of RPMI/5%FCS was added and then centrifuged at 400× *g* for 10 min, 4 °C. The pellet was resuspended in 2 mL of complete medium (RPMI-1640 supplemented with 50 µM β-mercaptoethanol, 1 mM glutamine, 1% penicillin-streptomycin, 1% NEAA, 1% sodium pyruvate and 10% FCS) for cell counting for cytometry analyses.

#### 4.7.2. Mediastinal Lymph Nodes (mLN) and Lymph Nodes (LN)

mLN and LN (inguinal and axillary) were collected separately and crushed with the help of a sterile syringe through 100 µm strainer in 3 mL of RPMI/5% FCS. The suspension was then centrifuged at 400× *g*, 4 °C for 8 min. The mLN and LN pellets were resuspended in 600 µL and 1 mL of complete medium for counting cells and for cytometry analyses, respectively.

#### 4.7.3. Bronchoaveolar Lavage Fluids (BALf)

BALf was removed from the lungs by three consecutive expulsions/aspirations of 1 mL of PBS in the airway compartment via a thin cut realized in the upper part of the trachea. The lavage was then centrifuged at 400× *g* for 10 min, 4 °C. The pellet was resuspended in 500 µL of RBC lysis buffer and incubated for 2 min at RT. Nine mL of RPMI/5%FCS were added for centrifugation at 400× *g* for 5 min, 4 °C. The pellet was then resuspended in 200 µL of complete medium and stored on ice until staining.

#### 4.7.4. Spleen

Splenocytes were prepared by passing through the spleen on a 70 µm sieve with the help of a sterile syringe in 6 mL RPMI/5% FCS. The cell suspension was then centrifuged at 400× *g*, 4 °C for 10 min. Then, splenocytes were treated as described for the lung.

#### 4.7.5. Blood

Whole blood from infected or control mice obtained by cardiac puncture was mixed with a solution containing 1.5 mM EDTA and 2% dextran and incubated at 37 °C for 30 min. Leukocyte-rich plasma, located on the top of the aggregated erythrocytes, was carefully collected and centrifuged at 400× *g* for 10 min at 4 °C. The cell pellets were mixed with 3 mL RBC lysis buffer for 3 min and washed with RPMI-1640 containing 5% FCS. Cells were stained for flow cytometry analysis.

#### 4.7.6. Intracellular Staining

For basal cytokines detection, lung suspensions were cultured at 1 × 10^6^ cells/mL in a 6/well plate and stimulated with 50 ng/mL PMA (Sigma-Aldrich, Saint Quentin Fallavier, France) and 5 μg/mL ionomycin (Sigma-Aldrich, Saint Quentin Fallavier, France) in the presence of brefeldin A (1 μL of GolgiPlug, BD Biosciences in 1 mL of complete RPMI medium). After 4 h of incubation at 37 °C with 5% CO_2_, cells were washed twice with PBS and incubated in FC blocker solution (2.4G2 FcR Block, produced in our laboratory) during 15 min on ice. Cells were then stained with surface antibodies including anti-CD3, CD4, CD8, CD62L, CD69, CD103 and CD44; then, they were washed twice with PBS and stained with viability dye (eFluor-506, Thermofisher, Illkirch, France). Before staining with intracellular or transcriptional antibodies against IFN-γ, IL-17A, IL-10, IL-2, IL-4, TNF-α and RORγt, cells were treated with BDcytofix solution and washed by Perm/Wash solution (both from BD biosciences, Le Pont de Claix, France) according to the manufacturer’s protocol. Finally, cells were washed with Perm/Wash and resuspended in PBS for FACS analysis.

BrdU incorporation was detected using the BrdU Flow kit (n°552598, BD Biosciences Le Pont de Claix, France) according to the manufacturer’s protocol. First, single-cell suspensions were isolated from the tissue of mice received FTY720/Brdu treatment. After staining of surface antigens, cells were treated with Cytofix/Cytoperm buffer for 30 min at RT. Cells were washed with Perm/Wash Buffer and fixed with Cytoperm Permeabilization Buffer Plus for 15 min. Cells were again washed with Perm/Wash buffer and were treated with Cytofix/Cytoperm for 10 min. Then, cells were incubated with DNase I (0.33 mg/mL in PBS) for 1 h at 37 °C before cells were stained with an antibody mixture for BrdU and other intracellular markers in Perm/Wash Buffer for 30 min at RT. Cells were then washed twice with Perm/Wash Buffer and resuspended in PBS for FACS.

### 4.8. FACS Analyses

All antibodies used for flow cytometry were purchased from eBioscience, BD Biosciences or BioLegend. Dead cells were discriminated in all experiments using Live/dead (BV-510) fixable dead stain (Thermofisher, Illkirch, France). All stains were carried out with FC-block. Cell suspensions were stained 20–30 min on ice by the following mABs: CD3e-eFlour450 (17A2), CD335-APC/eFlour780 (29A1.4), Rorγt-APC (B2D), CD8-BV711 (53-6.7), CD19-APC/Cy7 (1D3), IFNγ-BV786 (XMG1.2), IL-4-Pe/Cy7 (11B11), CD4-BV605 (RM4-5), CD62L-Pe/Cy5 (MEL-14), CD44-Alexa700 (IM7), CD69-Pe/Cy7 or Pe/Cy5 (H1.2F3), CD103 (ItgαE)-PerCP/Cy5.5 (2E7), CD11b-APC/Cy7 (M1/70), TNFα-APC/Cy7 (MP6-XT22), IL-2-PE (JES6-5H4), IL-17-AF488 (TC11-18H10.1), IL-10-PE/Dazzle594 (JES5-16E3), CD90.1- APC (OX-7) and CD90.2-BV510 (30-H12). In all flow cytometric plots, doublets, dead cells and CD19+CD335+CD11b+ (dump channel) were excluded and the populations were gated as follows: CD4+T cells (CD3+CD4+), CD8+ T cells (CD3+CD8+), activated CD4+/CD8+T cells (CD3+CD4+CD44+CD62L-) and Trm (CD3+ CD4+/CD8+ CD62L-CD44+CD69+CD103+). Cell acquisition was performed on a LSR II UV analyzer (BD Biosciences, Le Pont de Claix, France) using FACSDiva software (BD Biosciences), and data were analyzed using FlowJo software version 10.

### 4.9. Cell Sorting and Adoptive Transfer

Lungs and MLN were removed from donor mice (CD90.2+). Cells were prepared as previously described above. T cells were enriched negatively by using Pan T kit supplemented with CD62L antibody (MiltenyiBiotec, Paris, France). After, CD69+CD103+ Trm and CD69+CD103- subsets were sorted on an ARIAIII-SORP (BD Biosciences). Cellular purity of separated populations was determined by flow cytometric analysis and was routinely >97%. Sorted T cell subsets were prepared approximately 0.5–1.5 × 10^4^ cells in 10 µL of PBS and given intra-tracheally, or 1 × 10^6^ cells in 100 µL (Tnv), or 1 × 10^4^ cells in 100 µL (mLN) intra-venously to recipient mice (CD90.1+/CD90.2+). Three days later, the recipient mice were infected with SM as described above and euthanized at day 6 p.i. CFU counts were performed to access bacterial clearance and transferred cells were confirmed by FACS.

### 4.10. Immunostaining and FISH

Perfused lungs were incubated for 4 h at 4 °C in Antigenfix solution (P0014, Diapath, Martinengo, Italia). Lungs were dehydrated overnight in 30% sucrose solution and then frozen at −80 °C. Slices of 20 µm were used for immunofluorescence or FISH staining. Trm were stained following the protocol described in Lelouard et al., 2012 [43] with the following panel: CD4-eF450, CD8α-AF488, CD69-AF568, CD103-AF647 or CD4-eF450, CD69-AF488, and CD103-Cya3. FISH protocol was done as described in (Pédron et al., 2012) [44], and the Eub338-Alexa-555 probe 5′-GCTGCCTCCCGTAGGAGT-3′was used to stain bacteria in the lung. All the images were taken on LSM 780 (Zeiss, Marly le Roi, France) confocal microscope. Series of Z section were taken for each slides. Images were analyzed using Imaris 6.1.

### 4.11. Statistical Analysis

Statistical analyses were performed with GraphPad Prism software. The error bars show the standard deviation. For all the tests, we performed a Shapiro–Wilk test to assess for normality of data distribution. In case of normality, (i) when only two conditions were tested, we performed Welch *t*-test (since the validity of homoscedasticity hypothesis was never reached); (ii) when more than two conditions were compared, we performed a parametric one-way ANOVA, followed by Tukey test to assess the significance among pairs of conditions. In case of non-normality, (i) when only two conditions were tested, we performed a Mann-Withney U test; (ii) when more than two conditions were compared, we performed a non-parametric one-way ANOVA, followed by Dunn test to assess the significance among pairs of conditions.

## 5. Patents

IMPROVED PERTUSSIS VACCINE COMPOSITION: Ref: BEP180838 EP Patent n° 18306831.1-1116.

## Data Availability

The data presented in this study are available on request from the corresponding author. The data are not publicly available due to the patent (BEP180838 EP Patent n° 18306831.1-1116).

## Supplementary Materials

The following are available online at https://www.mdpi.com/article/10.3390/toxins13090632/s1, Figure S1: CD4+ CD69+CD103+ T cells subset is restricted to the lung. Figure S2: Pertussis Toxin (PTX) is required to generate CD8+ Trm subset in the lung of infected mice. Figure S3: CD4+ and CD8+ Trm subsets proliferate in the lung and are located at the front line of defense against *B. pertussis*. Figure S4: Cytokines production profiling of activated and Trm CD4^+^ T cells. Figure S5: Pertussis Toxin Ptx promotes γδ Trm cells and helps the bacteria to better colonize the lung of infected mice. Figure S6: Pertussis Toxin PTX is critique to generate Trm populations.
