# Supplementary material for: PTX Instructs the Development of Lung-Resident Memory T Cells in Bordetella pertussis Infected Mice
Source: Toxins (Basel). 2021 Sep 8;13(9):632. doi: 10.3390/toxins13090632 (PMC8470914; doi:10.3390/toxins13090632)
Supplement: Supplementary file 1 [file toxins-13-00632-s001.zip › toxins-1281668-supplementary.pdf]

Supplementary Materials: PTX Instructs the Development of Lung-Resident Memory T Cells in *Bordetella pertussis* Infected Mice

Julie Tomas, Yoon Koo, Dimitri Popoff, Vilma Arce-Gorvel, Sean Hanniffy, Jean-Pierre Gorvel and Cyrille Mionnet

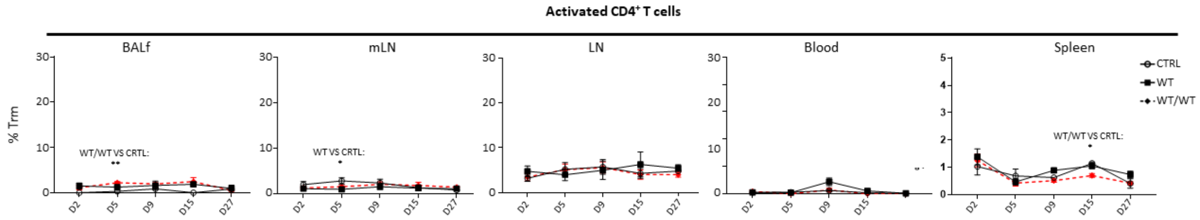

Figure S1. CD4+ CD69+CD103+ T cells subset is restricted to the lung. Representative flow cytometry of CD69 and CD103 expression of activated CD4+ T cells harvested from BALf, mLN, LN, blood and spleen of mice primary and secondary infected with WT strain day 15 p.i. * *p*-value < 0.05 WT/WT vs CTRL, ΔPTX /ΔPTX and ΔPTX /WT; ** *p*-value < 0.01 WT/WT vs CTRL, ΔPTX /ΔPTX and ΔPTX /WT.

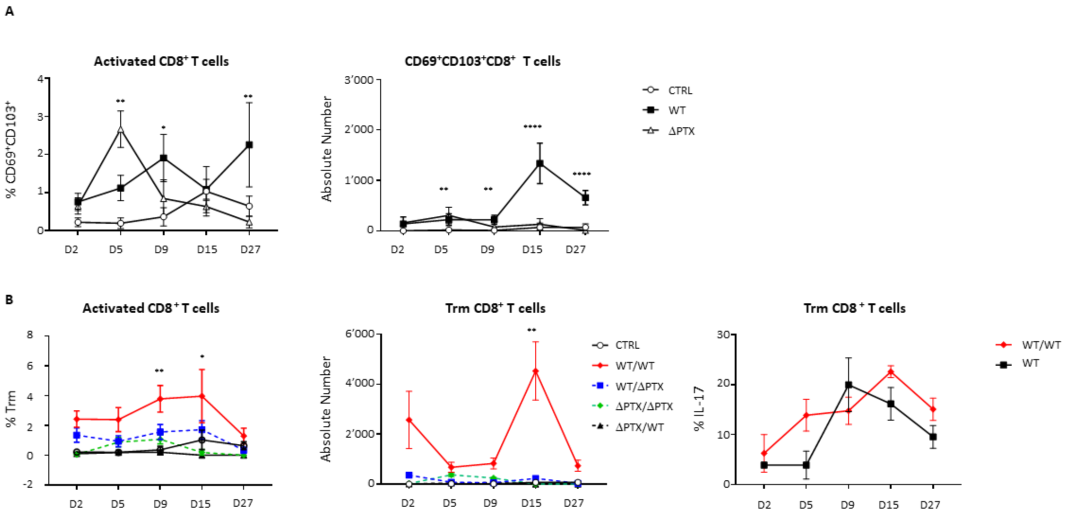

Figure S2. Pertussis Toxin (PTX) is required to generate CD8+ Trm subset in the lung of infected mice. Frequency and absolute number of CD8+ CD69+CD103+ T cells subset harvested from lung of mice primary (**A**) and secondary (**B**) infected with WT or ΔPTX strains. (C) Frequency of IL-17 producing CD8+ Trm cells at various times p.i in WT and WT/WT groups. (Results are mean ± SD for at least 3 to 5 mice/group and are representative of three independent experiments. The statistical significance of differences are shown. * *p*-value < 0.05 WT/WT vs CTRL, ΔPTX /ΔPTX and ΔPTX /WT; ** *p*-value < 0.01 WT/WT vs CTRL, ΔPTX /ΔPTX and ΔPTX /WT, *** *p*-value < 0.001 WT/WT vs CTRL, ΔPTX /ΔPTX and ΔPTX /WT, **** *p*-value < 0.0001 WT/WT vs CTRL, ΔPTX /ΔPTX and ΔPTX /WT.

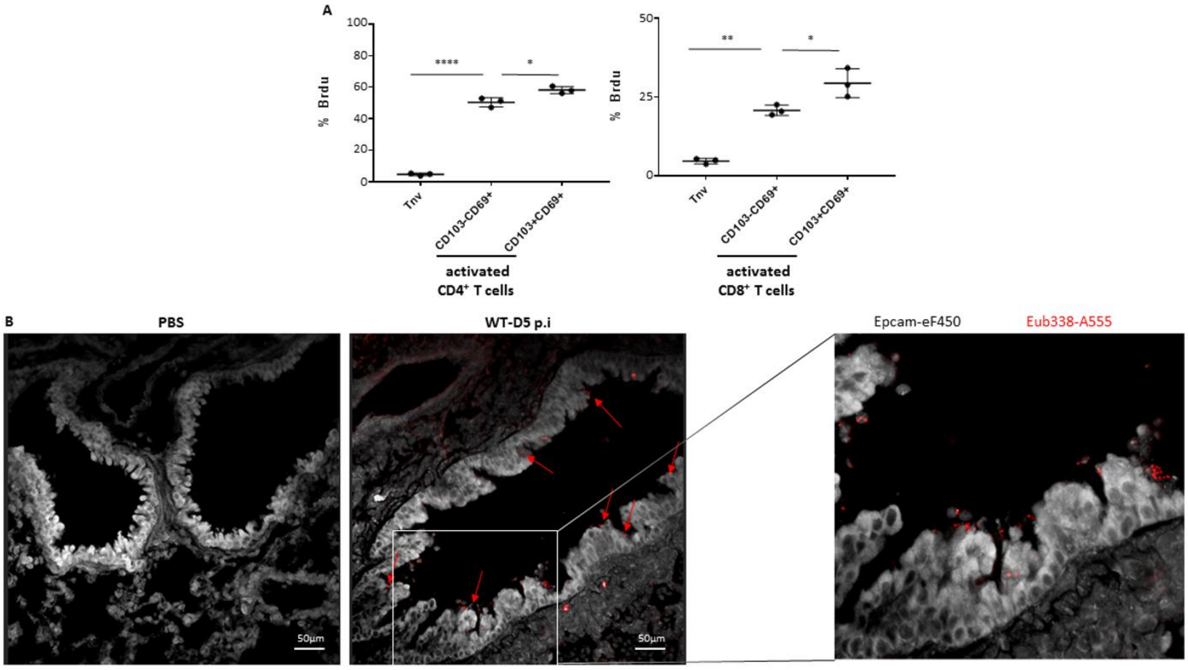

Figure S3. CD4+ and CD8+ Trm subsets proliferate in the lung and are located at the front line of defense against *B. pertussis*. (**A**) Frequancy of (**B**) Frequency of BrdU-expressing naïve T cells CD4+/CD8+ CD62L+CD44- T cells (Tnv), activated T cells CD4+/CD8+ CD62L-CD44+CD69+CD103- and CD4+/CD8+ CD69+CD103+ Trm harvested from the lung after treatment with FTY720 in WT/WT group at day 5 p.i. Results are mean ±SD for at least 3-4 mice/group and are representative of three independent experiments. Statistical significance of differences are shown. * p-value < 0.05; ** p-value < 0.01, **** p-value < 0.0001. (B) Representative micrographs of Fluorescent in situ Hybridization technique (FISH) showing the bacteria stained by the Eub338 probe (red) and the lung epithelium stained by the Epcam protein (grey). Groups showed are PBS (CTRL group), WT infected groups at day 5 pi.

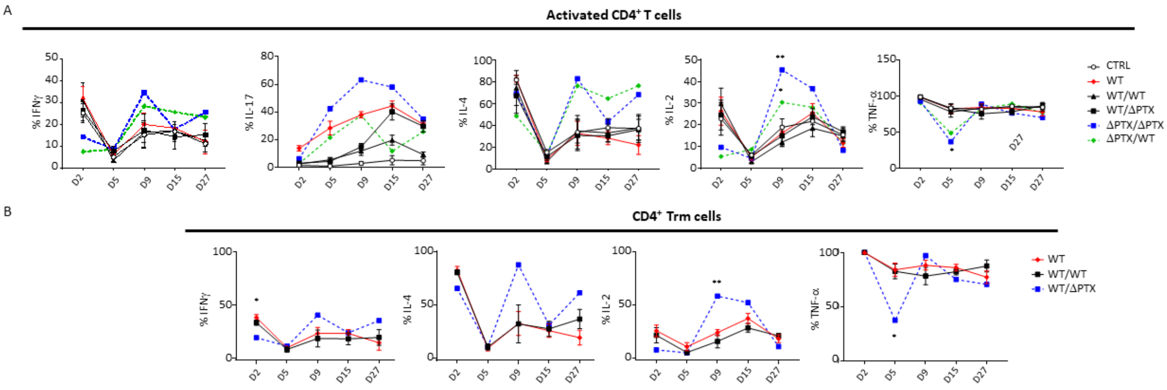

Figure S4. (**A**) Cytokines production profiling of activated and Trm CD4+ T cells**.** Frequency of IFN-γ, IL-17, IL-4, TNF-α, IL-2-producing activated CD4⁺ T cells at various times p.i in CTRL, WT, WT/WT, WT/ΔPTX, ΔPTX/ΔPTX and ΔPTX/WT groups. (**B**) Frequency of IFN-γ, IL-4, IL-2-and TNF-α-producing CD4⁺Trm at various time p.i. Results are mean ± SD for at least 3 to 5 mice/group and are representative of three independent experiments. The statistical significance of differences are shown. * p-value < 0.05; ** p-value < 0.01.

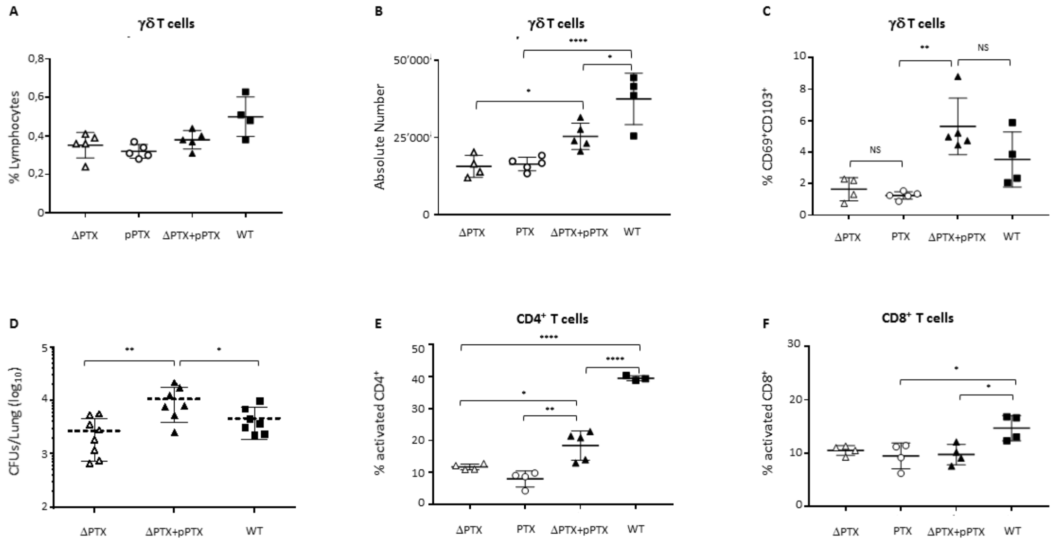

Figure S5. Pertussis Toxin Ptx promotes γδ Trm cells and helps the bacteria to better colonize the lung of infected mice. (**A**) Frequency and (**B**) absolute numbers of γδ T cells harvested from the lung of ΔPTX, PTX, ΔPTX+pPTX and WT infected mice at day 15 p.i. (**C**) Frequency of resident CD69+CD103+ γδ T cells. (**D**) Numbers of CFU of *B. pertussis*, enumerated at day 15 p.i harvested from the lung of ΔPTX, ΔPTX+pPTX and WT infected mice. (**E**) Frequency of activated CD4+ and (**F**) CD8+ harvested in the lung of ΔPTX, pPTX, ΔPTX+pPTX and WT.

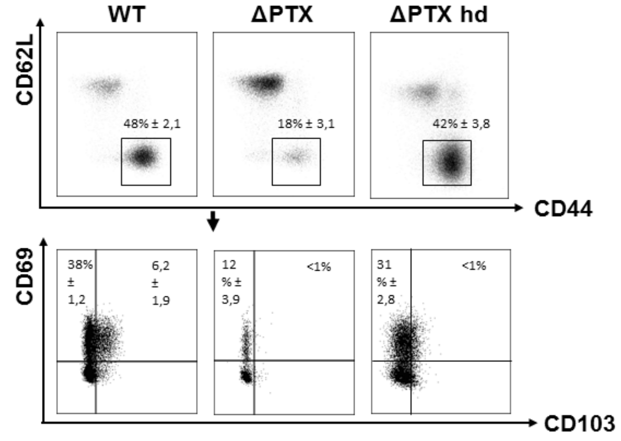

Figure S6. Pertussis Toxin PTX is critique to generate Trm populations. Mice were intra-nasally inoculated with 6x10^{6} CFU/20µL of WT or ΔPTX or with a high dose (60x10^{6} CFU) of ΔPTX of PBS (ΔPTXhd group). Gating Strategy, and frequency of activated CD4^{+} and CD4^{+}Trm populations recorded in the lung from D2 to D27 p.i. in primary infected mice. Here we show d15 p.i time point where the highest frequency of CD4^{+}Trm is obtained for WT infected mice. Results are mean for at least 4 to 5 mice/group.
